# Antibacterial, Antifungal, and Anticancer Effects of Camel Milk Exosomes: An In Vitro Study

**DOI:** 10.3390/vetsci10020124

**Published:** 2023-02-06

**Authors:** Amira M. Shaban, Mai Raslan, Zeina Walid Sharawi, Mohamed Sayed Abdelhameed, Ola Hammouda, Hossam M. El-Masry, Khaled N. M. Elsayed, Mohammed A. El-Magd

**Affiliations:** 1 Biotechnology & Life Sciences Department, Faculty of Postgraduate Studies for Advanced Sciences, Beni-Suef University, Beni-Suef 62511, Egypt; 2 Biological Sciences Department, Faculty of Sciences, King AbdulAziz University, Jeddah 21589, Saudi Arabia; 3 Botany and Microbiology Department, Faculty of Science, Beni-Suef University, Beni-Suef 62511, Egypt; 4 Chemistry of Natural and Microbial Products Department, Pharmaceutical and Drug Industries Research Institute, National Research Centre, Cairo 12622, Egypt; 5 Anatomy Department, Faculty of Veterinary Medicine, Kafrelsheikh University, Kafrelsheikh 33516, Egypt

**Keywords:** exosomes, camel milk, antibacterial, antifungal, anticancer

## Abstract

**Simple Summary:**

Camel milk (CM) and its exosomes (CM-EXO) have many health-promoting effects due to their antibacterial, antifungal, and anticancer effects. Herein, we investigated the CM-EXO antimicrobial effect on Gram-positive bacteria (*Staphylococcus aureus*, *Micrococcus luteus*, and *Enterococcus feacalis*), Gram-negative strains (*Escherichia coli*, *Pseudomonas aeruginosa*, and *Proteus mirabilis*), and *Candida albicans* and found only bacteriostatic effects against Gram-negative strains, and fungistatic effect. To the best of our knowledge, this is the first study to report a selective apoptotic effect of CM-EXOs on HepG2 and CaCo2 cells, but not on normal Vero cells. CM-EXOs also induced the elevation of intracellular reactive oxygen species and reduced antioxidant status in cancer cells but not in normal cells.

**Abstract:**

Camel milk (CM) has potent antibacterial and antifungal effects and camel milk exosomes (CM-EXO) have been shown to inhibit the proliferation of a large variety of cancer cells including HepaRG, MCF7, Hl60, and PANC1. However, little is known regarding the effects of CM-EXO on bacteria, fungi, HepG2, CaCo2, and Vero cells. Therefore, this study aimed to evaluate the antibacterial, antifungal, and anticancer effects of CM-EXO. EXOs were isolated from CM by ultracentrifugation and characterized by transmission electron microscope and flow cytometry. Unlike CM, CM-EXO (6 mg/mL) had no bactericidal effects on Gram-positive bacteria (*Staphylococcus aureus*, *Micrococcus luteus*, and *Enterococcus feacalis*) but they had bacteriostatic effects, especially against Gram-negative strains (*Escherichia coli*, *Pseudomonas aeruginosa*, and *Proteus mirabilis*), and fungistatic effects on *Candida albicans*. HepG2, CaCo2, and Vero cells were respectively treated with CM-EXOs at low (6.17, 3.60, 75.35 μg/mL), moderate (12.34, 7.20, 150.70 μg/mL), and high (24.68, 14.40, 301.40 μg/mL) doses and the results revealed that CM-EXOs triggered apoptosis in HepG2 and CaCo2 cells, but not in normal Vero cells, as revealed by high *Bax* expression and caspase 3 activities and lower expression of *Bcl2*. Interestingly, CM-EXOs also induced the elevation of intracellular reactive oxygen species and downregulated the expression of antioxidant-related genes (*NrF2* and *HO-1*) in cancer cells but not in normal cells. CM-EXOs have antibacterial and antifungal effects as well as a selective anticancer effect against HepG2 and CaCo2 cells with a higher safety margin on normal cells.

## 1. Introduction

Camel milk (CM) has immunostimulant, antioxidant, anticancer, antibacterial, antifungal, antiviral, and hypoglycemic effects [1,2]. Unlike other ruminants, CM is rich in vitamin C, minerals (sodium, potassium, sodium, iron, zinc, copper, and magnesium), and immunostimulant/antibacterial proteins (lysozyme, immunoglobulins, lactoferrin, lactoperoxidase, and caseins), but is poor in cholesterol and sugar [3,4]. Lactoferrin can serve as a bacteriostatic and/or bactericidal agent on Gram-positive and Gram-negative bacteria [5]. Camel colostrum is rich in lactoferrin and has a bactericidal potential against *E. coli* and a bacteriostatic effect against *L. monocytogenes*, whereas CM from other lactation stages only has bacteriostatic potential against these two bacteria [6]. CM lactoperoxidase has a bactericidal potential against Gram-negative bacteria and a bacteriostatic effect against Gram-positive bacteria [5], while CM caseins have bactericidal and bacteriostatic effects on both bacteria [7]. CM possesses antibacterial properties against *S. aureus* and *E. coli* and has a synergistic effect with antibiotics, which means it may be used to lower drug doses and minimize bacterial antibiotic resistance [8]. Moreover, CM supplementation was shown to reduce oxidative stress and restored antioxidant enzymes activities, which were deteriorated by injections of *E. coli* and *S. aureus* [9].

CM also has anticancer potential against a large variety of malignancies including hepatocellular carcinoma, colon carcinoma, breast cancer, glioma, lung cancer, and leukemia [2,10]. Liver cancer progression can be inhibited by CM as this milk contains strong antiviral and anti-oxidative constituents in addition to highly active antibodies that could bind to and destroy tumor cells without harming normal cells [11]. CM may also have thrombolytic properties since it inhibits coagulation and fibrin production, which slows the spread and proliferation of metastatic tumor cells [12]. Lactoferrin, a significant iron-binding protein found in CM, has been demonstrated to lower the risk of cancer by 56% [13]. Korashy et al. [14] studied the molecular mechanisms governing CM’s effect on human cancer cells and found that it causes apoptosis in HepG2 and MCF7 cells via oxidative-stress-mediated processes and apoptotic processes.

Exosomes (EXOs) are membrane nanovesicles with a diameter of 40–150 nm. [15]. They are made by a set of cells with varied activities [16], and have been found in a variety of biological fluids [17] including milk [18]. EXOs contain a diversity of biomolecules, including nucleic acids such as miRNA, short noncoding RNA, and mRNA, that represent their biological origin [19]. Their nucleic acids can be translated into functional proteins or influence gene activity. Commercial milk has stable EXOs that remain intact in the gastrointestinal system and have an immunoregulatory impact [20]. Obesity, type 2 diabetes, cancer, and neurological illnesses are all linked to microRNA in cow milk [21]. Despite the presence of RNAase activity, these physically stable vesicles may function as cargo for diverse RNA types in bovine milk, potentially causing trans-species transcriptome regulation [22]. CM-EXOs could help people recover from a variety of ailments [23,24]. CM-EXOs have anticancer properties against HepaRG, MCF7, Hl60, and PANC1, perhaps by triggering apoptosis and reducing inflammation, oxidative stress, metastasis, and angiogenesis [10,23,25,26,27]. Recently, Tong et al. [28] reported that administration of cow milk extracellular vesicles (including exosomes) to mice modulated gut microbiota with increased count of some classes of bacteria related to the maintenance of gut health and improved gut immune response.

As previously mentioned, CM has potent antibacterial, antifungal, and anticancer effects and its exosomes (CM-EXOs) inhibit the proliferation of HepaRG, MCF7, Hl60, and PANC1. However, there is a shortage in the available literature data regarding the effects of CM-EXO on bacteria, fungi, HepG2, and CaCo2 cells. Thus, this study was conducted to determine the impact of CM-EXOs on various bacteria, fungi, and these two cell lines.

## 2. Materials and Methods

### 2.1. Animals and Milk Sampling

Twenty camels (*Camelus dromedaries*) were used in this study, all of which were kept in good health on a national local farm in Marsa Mattrouh, Egypt. Ages of these animals ranged from 8 to 12 years, and they had at least three full lactation cycles. All milk samples were collected at the mid-lactation period under a complete aseptic condition to minimize bacterial contamination. In order to isolate the exosomes as soon as possible, milk samples were transported in an icebox to the laboratory on the same day.

### 2.2. Exosome Isolation and Characterization

Differential ultracentrifugation was used to isolate the exosomes. Fat globules, casein aggregates, and other debris were removed from the milk sample by centrifugation at 5000 g for 15 min at 4 °C then at 13,000 g/30 min/4 °C. Exosomes were extracted from supernatants by twice ultracentrifugation at 100,000 g (Optima L-90okay; Beckman Coulter) for 90 min each at 4 °C, followed by a brief wash with phosphate-buffered saline (PBS) to remove large debris and microvesicles. To produce a homogeneous suspension, the exosome pellets were collected and resuspended in PBS at a concentration of 6 mg/mL, centrifuged at 5000 rpm for 30 min twice with the 100 kDa filters, and stored at 4 °C until the next use. Transmission electron microscopy (TEM) at 80 kV was used to identify the size of exosomes as previously described [27]. Confirmation of exosomal isolation was carried out by the detection of specific exosomal proteins CD63 and CD81 using anti-CD63 (1:200, Santa Cruz, Germany) and anti-CD81 (1:200, Santa Cruz, Germany) by Attune flow cytometer (Applied Biosystem, Foster City, CA, USA) and a standard Nanobead calibration kit containing beads (50 and 100 nm, Technologies Drive Fisher, Ann Arbor, MI, USA).

### 2.3. Microbial Analysis

We investigated the effects of CM or CM-EXOs on Gram-positive bacteria (*Micrococcus luteus* (ATCC 10240)*, Staphylococcus aureus* (ATCC 6538) and *Enterococcus faecalis* (ATCC 29212)), Gram-negative bacteria (*Escherichia coli* (ATCC 25922), *Pseudomonas aeruginosa* (ATCC27853), and *Proteus mirabilis* (ATCC 9240)), and pathogenic yeast (*Candida albicans* (ATCC 10231)). The inoculum size of these pathogenic strains was prepared and adjusted to 0.5 McFarland standard (1.5 × 10^8^ CFU/mL). The bacterial or fungal suspension (25 µL) was inoculated into a plate containing 20 mL of the sterile nutrient agar (NA) medium. After the media cooled and solidified, the prepared sample was divided into 2 groups, the first one was applied on a 0.9 cm well of that inoculated agar plates prepared previously, each well was filled with 100 µL of the sample while the second group was used in the shake flask method applied in a 100 mL conical flask containing 20 mL of nutrient broth medium (NB) inoculated with 25 µL bacterial and fungal suspensions and treated with 100 µL of the sample. The concentration of CM-EXOs was 6 mg/mL. The seeded plates were chilled for one hour before being incubated at 37 °C for 24 h, and the zones of inhibition (ZI) were measured in millimeters. The shake flask method was used to calculate the antimicrobial activity expressed throughout the reduction of the bacterial count (%) using the turbidimetric measurement technique to calculate the colony-forming unit (CFU) of these tested strains after treatments compared to the number of microorganisms surviving in the control flask after 24 h of incubation at 37 °C. All results were expressed according to the following equation: Relative [OD Reduction (%)] = (A- B/A) × 100 where, A: the number of microorganisms present on the control flask contains pathogenic strains only without any treatment, B: the number of microorganisms present in tested flasks after applying the tested sample treated.

### 2.4. Cytotoxicity by MTT Assay

Human hepatocarcinoma (HepG2), colorectal adenocarcinoma (CaCo2), and normal kidney cell lines (Vero) were purchased from VACSERA (Egypt). MTT (Molecular Probes, Eugene, OR, USA; Cat.no.V-13154) was used to test the antiproliferative effect of CM-EXOs on the three cell lines. The cells were suspended in Dulbecco’s Modified Eagle’s medium (DMEM, GIBCO, Grand Island, NY, USA; Cat. no.11995073) supplemented with 10% heat-inactivated fetal bovine serum (GIBCO, Grand Island, NY, USA, Cat. no.10099133). CM-EXOs and the standard anticancer drug cisplatin were separately applied to the wells containing seeded cells at a density of 1 × 10^4^ cells/well (100 μL/well) to achieve final concentrations of CM-EXOs and cisplatin ranging from 0 to 200 and 0 to 50 μg/mL for HepG2 and CaCo2 cells and from 0 to 500 and 0 to 50 μg/mL for Vero cells, respectively, and the cells were cultured for 24 h. Each well received 10 μL of 12 mM MTT stock solution (5 mg/mL MTT in sterile PBS) after the incubation period. The plate was then incubated at 37 °C for 4 h. The purple formazan crystal generated at the bottom of the wells was dissolved in 100 μL dimethyl sulfoxide (DMSO, Sigma Aldrich, St. Louis, MO, USA; Cat no.673439) for 20 min after the MTT solution was withdrawn. The absorbance was measured at 570 nm and the results were plotted using sigmoidal using GraphPad Prism 7 statistic software to calculate the IC_50_.

### 2.5. Experimental Design

Cells were divided into 4 groups: the Cnt group (untreated control cells), the EXO-L group (cells treated with low dose (¼ IC_50_) of CM-EXOs equivalent to 6.17 μg/mL in HepG2, 3.60 μg/mL in CaCo2, 75.35 μg/mL in Vero), the EXO-M group (cells treated with moderate dose (½ IC_50_) of CM-EXOs equivalent 12.34 μg/mL in HepG2, 7.20 μg/mL in CaCo2, 150.70 μg/mL in Vero), and the EXO-H group (cells treated with high dose (IC_50_) of CM-EXOs equivalent to 24.68 μg/mL in HepG2, 14.40 μg/mL in CaCo2, 301.40 μg/mL in Vero). All cells were then incubated for 24 hrs and then they were prepared for RNA isolation and biochemical assays.

### 2.6. Caspase 3 Activity Assay

Cells were lysed by RIPA lysis buffer and protein content was quantified by a BCA kit using BSA as a standard (Thermo Scientific, Rockford, IL, USA). The isolated protein (30 μg) was then incubated with 200 μM caspase 3 fluorogenic substrate (Ac-DEVD-AMC, Alexis Biochemicals, San Diego, CA, USA) and a fluorescent microplate reader observed the caspase 3 substrate cleavage at 405 nm.

### 2.7. Detection of Intracellular Reactive Oxygen Species

Analysis of intracellular reactive oxygen species (ROS) was performed fluorometrically by monitoring the conversion of the non-fluorescent probe 2,7-dichlorofluorescein diacetate (DCF-DA) to its fluorescent metabolite dichlorofluorescein (DCF). In brief, cells that had been cultured in 96-well cell culture plates and grew to 90% confluence were subjected to treatment with various concentrations of CM-EXOs for 24 hrs. After being washed twice with PBS, the cells were treated with 10 µM DCF-DA in fresh medium for 30 min. Through the use of a fluorescent microplate reader, we were able to directly measure the fluorescence at 485 nm for excitation and 535 nm for emission.

### 2.8. Gene Expression Analysis by qPCR

The relative expression of apoptosis-related genes (*Bax* and *Bcl2*), and antioxidant-related genes (*NrF2* and *HO-1*) after treatment with CM-EXOs at different concentrations were determined in the three cell lines by qPCR. We first extracted total RNA using a Trizol reagent (Invitrogen, Carlsbad, CA, USA, Cat# 15596026) according to the manufacturer’s instructions. RNA concentration and purity were detected by a Nanodrop (Q5000, Quawell, San Jose, CA, USA). Next, cDNA was obtained by reversing a transcription kit (Applied Biosystems, Waltham, MA, USA) using 5 μg RNA. Finally, a PCR mixture (2 μL cDNA, 1 μL from each primer, 12.5 μL 2 X SYBR Green Mix, and 8.5 μL RNase-free water) was run in the Step One Plus thermal cycler (Applied Biosystem, Waltham, MA, USA). The thermal cycles were carried out as previously described [10] and *β actin* was used as an internal control. Sequences of all used primers were presented in Table 1. Gene expression was shown in the form of fold change mean ± standard error of the mean (SEM) and was calculated by the 2^-∆∆Ct^ method.

### 2.9. Statistical Analysis

The variance between the groups was determined using a one-way analysis of variance using GraphPad Prism 7. Tukey’s honestly significant difference test was used to compare the means. The data were given as mean + standard error of the mean (SEM) with *p* ≤ 0.05 denoting significance.

## 3. Results

### 3.1. Characterization of Exosomes Derived from Camel Milk

**Figure 1:**
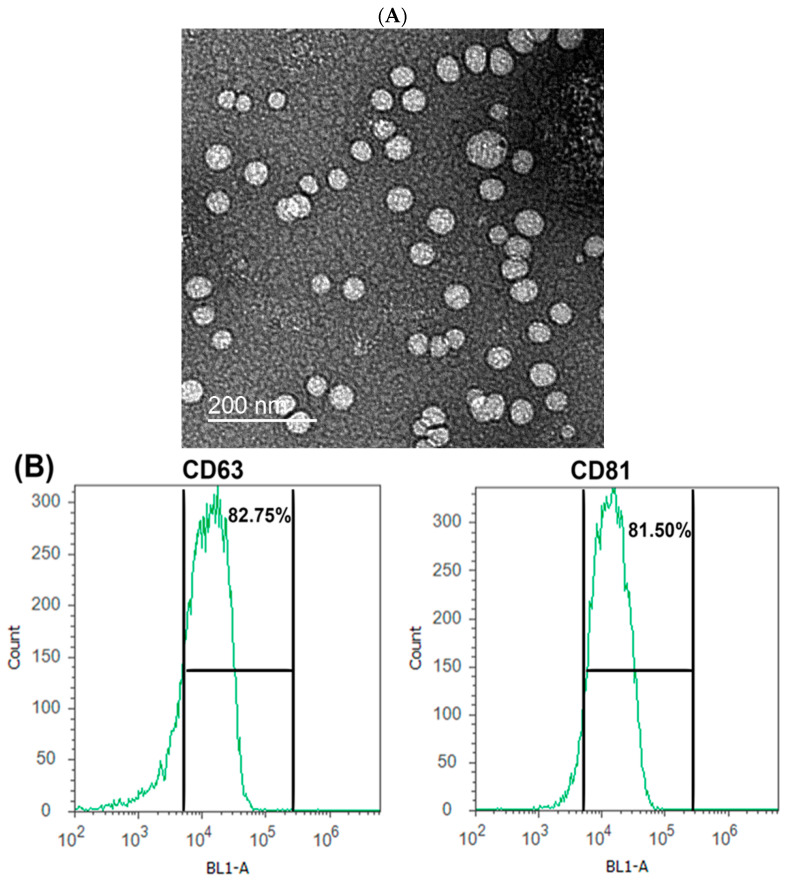
Characterization of EXOs obtained from CM. (**A**) TEM photograph for CM-EXOs, Scale bar = 200 nm. (**B**) The percentage of exosomal CD63 and CD81 protein positivity as determined by flow cytometry analysis.

As examined by transmission electron microscopy (TEM), CM-EXOs appeared as nanovesicles with typical diameters ranging from 30 to 100 nm (Figure 1A). Exosomal marker proteins CD63 and CD81 were detected at relatively high levels (82.75% and 81.50%, respectively) in flow cytometry analyses (Figure 1B). These findings inferred that exosomes were effectively isolated from the camel milk.

### 3.2. Antimicrobial Effect of Camel Milk Exosomes

**Figure 2:**
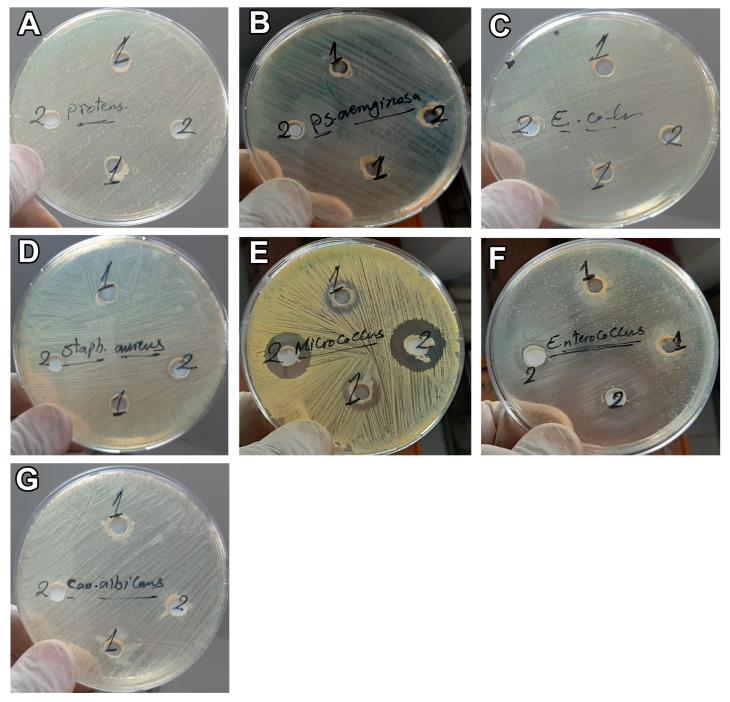
Inhibition zone assay shows the effect of CM on Gram-negative bacteria: *P. mirabilis* (**A**), *P. aeruginosa* (**B**) and *E. coli* (**C**), Gram-positive bacteria: *S. aureus* (**D**), *M. luteus* (**E**), and *E. feacalis* (**F**), and fungi, *C. albicans* (**G**).

**Figure 3:**
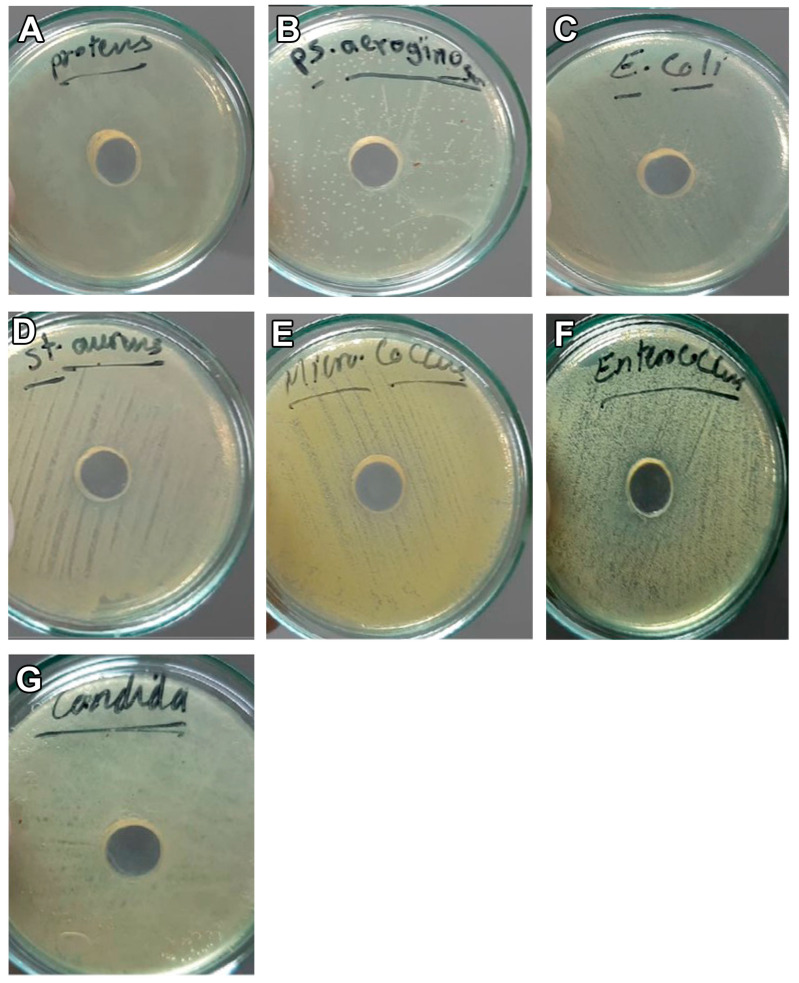
Inhibition zone assay shows the effect of CM-EXOs on Gram-negative bacteria: *P. mirabilis* (**A**), *P. aeruginosa* (**B**) and *E. coli* (**C**), Gram-positive bacteria: *S. aureus* (**D**), *M. luteus* (**E**), and *E. feacalis* (**F**), and fungi, *C. albicans* (**G**).

The results exhibited the presence of an inhibition zone for CM toward Gram-positive bacteria (*S. aureus*, *E. feacalis*, and *M. luteus*) with a size of 11, 16, and 27 mm, respectively (Table 2 and Figure 2 and Figure 3). However, no inhibition zone was noticed for CM on Gram-negative bacteria (*E. coli*, *P. aeruginosa*, and *P. mirabilis*) or *C. albicans* and no inhibition zone was observed after the addition of CM-EXOs to all the tested bacteria and yeasts (Table 2 and Figure 2 and Figure 3). On the other hand, the shake flask method revealed that the addition of CM resulted in a significant higher CFU reduction (%) in Gram positive bacteria and *C. albicans* than that observed after the addition of CM-EXOs (Table 3). In contrast, CM-EXO samples showed a significantly higher reduction in Gram negative bacteria than CM samples. These data infer that CM has the best bactericidal and bacteriostatic effects against Gram-positive strains but CM-EXOs had the superior bacteriostatic effect against Gram-negative strains.

### 3.3. CM-EXOs Cytotoxic Effect on Cancer Cells

**Figure 4:**
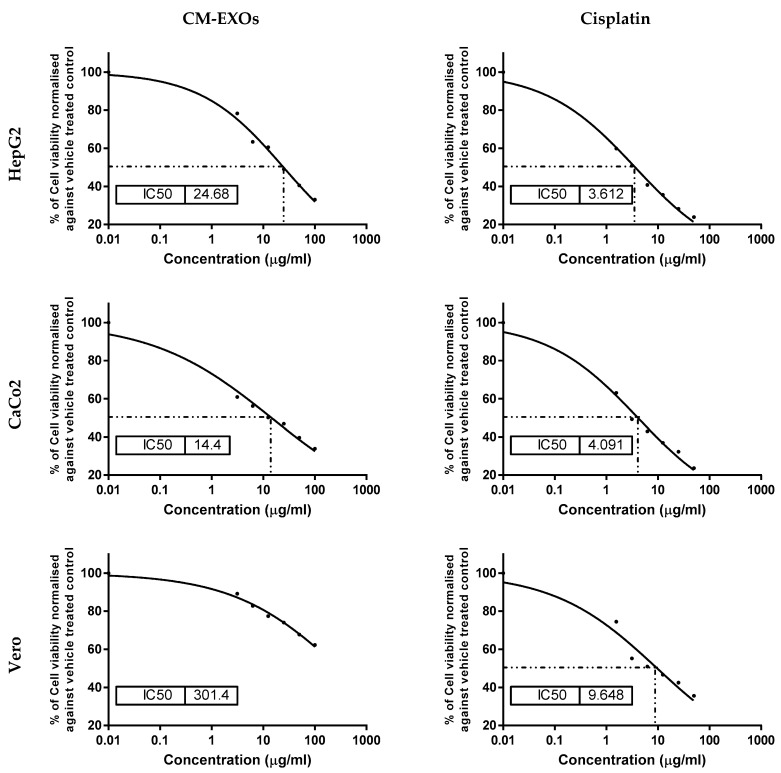
The sigmoidal curves for the effect of CM-EXOs and cisplatin on HepG2, CaCo2, and Vero cells show the IC_50_. Each data point represents an average of three independent experiments (*n* = 3).

The impact of CM-EXOs on the proliferation of human hepatocellular carcinoma cancer cells (HepG2), colorectal adenocarcinoma (CaCo2), and normal kidney (Vero) cell lines was assessed using the MTT assay (Figure 4). CM-EXOs were demonstrated to have potent anti-proliferation activity on HepG2 and CaCo2 cells with IC_50_ of 24.68 ± 1.67 and 14.40 ± 1.12 μg/mL, respectively, but with no cytotoxic potential on Vero cells as indicated by a very high IC_50_ value of 301.4 ± 16.42 μg/mL. On the other hand, the standard anticancer drug cisplatin showed a higher cytotoxic effect on both cancer (HepG2 and CaCo2) and normal (Vero) cells with IC_50_ of 3.61 ± 0.39, 4.09 ± 0.52, and 9.64 ± 0.74 μg/mL (Figure 4). These results infer that CM-EXOs had a lower cytotoxic effect against HepG2 and CaCo2 than cisplatin, but with a higher safety margin (less toxic potential) on the normal Vero cells than cisplatin.

### 3.4. CM-EXOs Inhibited Cancer Cell Proliferation via Apoptosis

**Figure 5:**
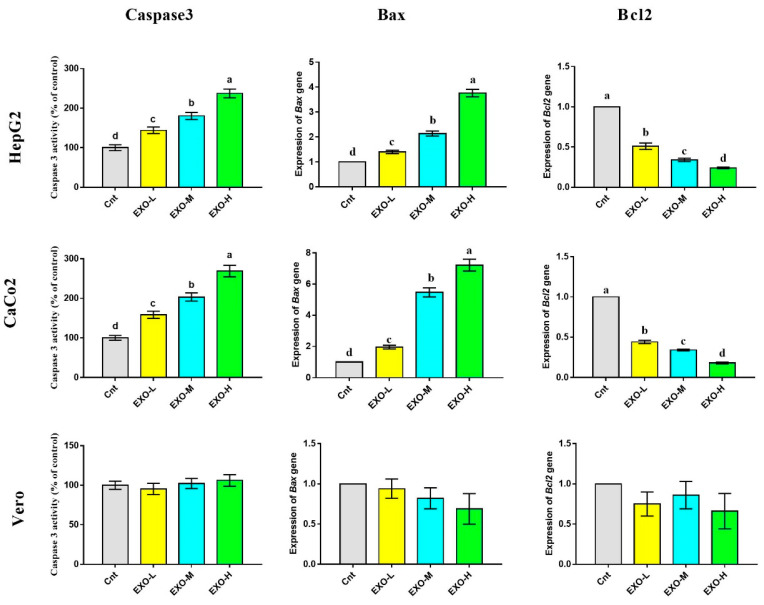
Effect of CM-EXOs on activities of caspase 3 and expression of *Bax* and *Bcl2* as detected by ELISA and qPCR, respectively. Values are expressed as fold change mean ± SEM (*n* = 3/group). Columns with different letters are significantly different at *p* < 0.05. All groups were compared to each other. Cnt, control cells; EXO-L, cells treated with low dose of EXOs; EXO-M, cells treated with medium dose of EXOs, and EXO-H, cells treated with high dose of EXOs.

As a final product of apoptosis, caspase 3 activity was determined by ELISA following treatment of HepG2, CaCo2, and Vero cells with CM-EXOs at three different concentrations (Figure 5). HepG2 and CaCo2 cells treated with CM-EXOs showed significantly higher caspase 3 activity than the control cells. This apoptotic effect was dose-dependent with highest activity in cells treated with a higher concentration of CM-EXOs (24.68 μg/mL for HepG2 and 14.40 μg/mL for CaCo2). The apoptotic potential of CM-EXOs was further confirmed at mRNA levels by the detection of the apoptotic gene *Bax* and the anti-apoptotic gene *Bcl2* in HepG2 and CaCo2 cells following the treatment with CM-EXOs (Figure 5 and Supplementary File). CM-EXOs administration resulted in a significant increase in *Bax* expression and a significant decrease in *Bcl2* expression in cancer cells relative to the control cells. Again, the change in *Bax* and *Bcl2* was dose-dependent with the best apoptotic effect for CM-EXOs at a higher dose. Unlike cancer cells, the normal Vero cells showed insignificant changes in caspase 3 activity and expression of *Bax* and *Bcl2* genes (Figure 5 and Supplementary File).

### 3.5. CM-EXOs Triggered Oxidative Stress in Cancer Cells

**Figure 6:**
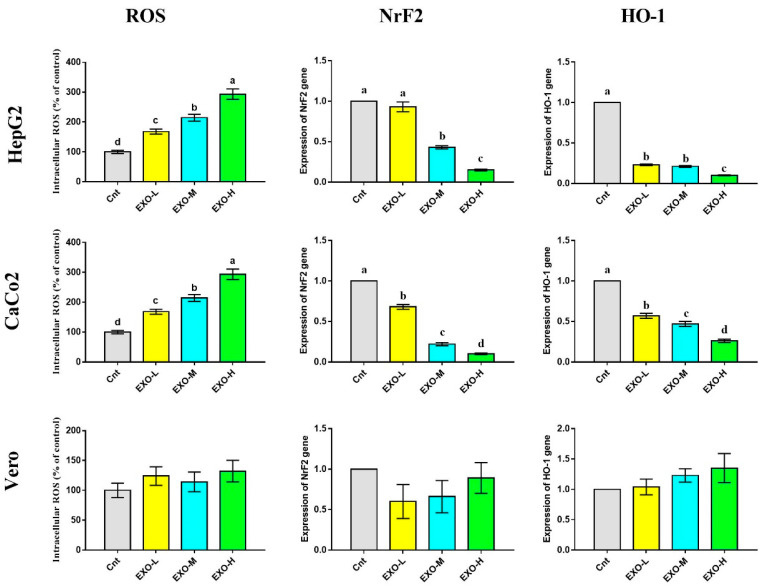
Effect of CM-EXOs on intracellular ROS level and expression of *NrF2* and *HO-1* as detected by ELISA and qPCR, respectively. Values are expressed as fold change mean ± SEM (*n* = 3/group). Columns with different letters are significantly different at *p* < 0.05. All groups were compared to each other. Cnt, control cells; EXO-L, cells treated with low dose of EXOs; EXO-M, cells treated with medium dose of EXOs, and EXO-H, cells treated with high dose of EXOs.

The CM-EXO effect on the intracellular ROS was investigated by ELISA and the results showed a significant dose-dependent elevation of the intracellular ROS following treatment of HepG2 and CaCo2 cells with CM-EXOs compared to the control cancer cells (Figure 6). In contrast, cancer cells treated with CM-EXOs exhibited significantly downregulated expression of antioxidant-related genes (*NrF2* and *HO-1*), with lowest expression in cells treated with highest dose of EXOs (EXO-H), relative to the control cells (Figure 6). On the other hand, the normal Vero cells did not exhibit any significant changes in the intracellular ROS and the expression of *NrF2* and *HO-1* genes (Figure 6 and Supplementary File).

## 4. Discussion

Oral administration of milk-derived EXOs has the potential to alter the gut flora of the host and can contribute to the interaction between gut bacteria and the host [28]. In vitro, the addition of milk EXOs to bacterial culture media induced bacterial growth [29]. Because changes in microbial populations can induce changes in the synthesis of microbial metabolites, gut microorganisms may operate as transmitters or amplifiers of dietary EXO signals [30]. Moreover, pathogens (such as fungi, viruses, and bacteria) can induce host cells to produce EXOs harboring relevant harmful components (such as polysaccharides, lipids, and glycosphingolipids) following infection [31]. EXOs then transmit these harmful substances to distant host cells, thereby participating in the spread of infection [32]. Therefore, the importance of EXOs in bacterial infection and the underlying processes have lately received a substantial amount of attention, and there are still several study mechanisms to be discovered. This study was conducted to evaluate the action of CM-EXOs on *Staphylococcus aureus, Micrococcus luteus, Enterococcus faecalis*, *Escherichia coli*, *Pseudomonas aeruginosa, Proteus mirabilis*, *Candida albicans*, and tumor cell lines (HepG2, and CaCo2) which mostly target the digestive system. We first extracted the EXOs from CM with diameters of 30–100 nm similar to those of CM-EXOs isolated by others [10,20,21,22,23,24,25,26,27], but disagreed with the milk EXOs of bovine (80–130 nm) [20] and buffalo (30–200 nm) [33]. The isolation process (ultracentrifugation vs. commercial kits), the type of milk (fresh vs. frozen), and species difference may all have a role in the size difference of EXOs.

In the current experiment, CM had bactericidal effects on Gram-positive bacteria (*S. aureus*, *M. luteus,* and *E. feacalis*) and *C. albicans* as revealed by the inhibition zone assay and the shake flask method. This bactericidal effect was attributed to the presence of abundant antimicrobial components including lactoperoxidase and lysozyme in CM [34]. In contrast, CM-EXOs had no bactericidal effects on Gram-positive bacteria but they had higher bacteriostatic effects against Gram-negative strains (*E. coli*, *P. aeruginosa*, and *P. mirabilis*), and higher fungistatic effects on *C. albicans* than the CM. EXOs derived from bovine milk change microbial populations in nonbovine animals, indicating that EXOs and their cargos are involved in bacterial–animal crosstalk [29]. Exosome-like vesicles originating from Apis mellifera, royal jelly, honey, and bee pollen had bactericidal, bacteriostatic, and biofilm-repressive performance on *S. aureus* [35]. Lactoferrin in CM prevents the growth of Gram-negative bacteria by binding iron and rendering it unavailable for bacterial growth and proliferation [36]. Electrostatic interactions between the negatively charged lipid bilayer and the positively charged lactoferrin surface create changes in membrane permeability, resulting in bactericidal activity [37]. Lactoferrin inhibits the formation of *P. aeruginosa* biofilms in vitro by inducing bacteria to migrate, preventing them from attaching to surfaces due to a shortage of iron in the environment [38]. Lactoferrin has antibacterial action against Gram-positive (*S. epidermidis*) and Gram-negative (*C. jejuni, Salmonella*) bacteria; however, it is more efficient against Gram-positive bacteria than Gram-negative bacteria [37]. CM is rich in lactoferrin, and it was recently proved that lactoferrin could be also expressed in CM-EXOs [24,27]. Therefore, the antimicrobial effects of CM and bacteriostatic effects of CM-EXOs could be attributed to the high quantity of lactoferrin in CM and CM-EXOs but this hypothesis should be first experimentally validated.

CM and CM-EXOs have anticancer action against a large variety of cancer cell lines including HepG2, HepaRG, MCF7, Hl60, and PANC1 [10,14,23,25,26,27,39,40,41]. However, no published research has evaluated the effect of CM-EXOs on HepG2 or CaCo2. This motivates us to investigate this effect and our results revealed a potent cytotoxic effect of CM-EXOs against the two cell lines with the best therapeutic effect against CaCo2. A potent anticancer agent might specifically target cancer cells while inflicting little or no harm to normal cells and having few or no side effects. In parallel, we did not find a cytotoxic effect on normal Vero cells after treatment with CM-EXOs. This suggests that CM-EXOs have a high selectivity for cancer cells as a target.

Cancer cells maintain their viability through the inhibition of apoptosis and reduction of intracellular ROS so any agent that could induce apoptosis and elevate ROS would kill these cells. We found that CM-EXOs triggered apoptosis in CaCo2 and HepG2, but not in normal Vero cells, as revealed by higher expression of the *Bax* gene and activities of caspase 3 and lower expression of the *Bcl2* gene. Our findings agreed with those obtained by other researchers who found that CM-EXOs induced apoptosis in HepaRG [27], MCF7 [10,25], Hl60 [23], and PANC1 [26]. In contrast, Ross et al. [42] reported that bovine-derived exosomes maintained CaCo2 as metabolically active and enhanced their viability in vitro but did not stimulate neoplastic development. The anticancer activity of CM and CM-EXOs could be attributed to lactoferrin which is abundantly found in CM [2,27]. Moreover, bovine lactoferrin can induce apoptosis and stop tumor growth in vitro, and it also inhibits malignant cells from progressing from the G1 to the S phase of the cell cycle [43].

Most of the chemotherapeutic agents depend on their action on the induction of oxidative stress inside cancer cells and tumor microenvironments [24]. We also found higher intracellular ROS and low expression of antioxidant-related genes (*NrF2* and *HO-1*) in cancer cells treated with CM-EXOs. These results indicate that CM-EXOs may have an inhibitory effect on HepG2 and CaCo2 cells by targeting the oxidative stress pathway. Consistent with these results, CM can elevate ROS and inhibit *HO-1* expression in hepatoma Hepa1c1c7 cells [14]. In contrast, Badawy, El-Magd and AlSadrah [10] reported lower levels of the lipid peroxidation biomarker (MDA), upregulated expression of *iNOS*, and higher activities of antioxidant enzymes (SOD, CAT, GPx) in tumor tissues of MCF7 xenograft following treatment of rats with CM-EXOs. This discrepancy in findings could be due to the different types of experiments conducted (in vitro against in vivo). On the other hand, no effect of CM-EXOs was noticed on the ROS/antioxidant status of normal Vero cells. In recent research, bovine milk EXOs were discovered to not only increase the proliferation of rat natural intestinal crypt epithelial cells (IEC-6) but also protect them from oxidative stress [44]. Additionally, human breast milk-derived EXOs can decrease epithelial cell mortality induced by oxidative stress [30,45]. We propose that exosomes, as mediators, follow distinct paths depending on whether they come from cancer cells or healthy cells. The role of EXOs in mediating cross-talk between healthy cells and cancer cells in the tumor microenvironment is still being investigated.

## 5. Conclusions

CM and CM-EXOs have a very positive impact on the treatment of harmful bacteria and fungi because they prevent their growth. CM-EXOs also have a beneficial impact on cancer treatment as revealed by their selective targeting of cancer cell lines (HepG2 and CaCo2) without any harmful effect on the normal cells (Vero). Induction of apoptosis and oxidative stress are among the two possible mechanisms by which CM-EXOs could inhibit cancer cell proliferation. The antibacterial, antifungal, and anticancer effects of CM-EXOs could be attributed to their molecular cargo, especially lactoferrin, which was recently shown to be abundantly expressed in CM-EXOs. However, further investigation is needed to understand the underlying mechanism by which CM-EXOs can exert this action.

## Figures and Tables

**Table 1:** Primers used for qPCR.

Gene	Forward (5^′^ ------ 3^′^)	Reverse (5^′^ ------ 3^′^)	Accession Number	Length (bp)
*Bax*	TGCTTCAGGGTTTCATCCAG	GGCGGCAATCATCCTCTG	NM_001291428.2	170
*Bcl2*	GAACTGGGGGAGGATTGTGG	CATCCCAGCCTCCGTTATCC	NM_000633.3	164
*NrF2*	CAGCGACGGAAAGAGTATG	TGGGCAACCTGGGAGTAG	NM_006164.5	200
*HO-1*	CGGGCCAGCAACAAAGTG	AGTGTAAGGACCCATCGGAGAA	NM_002133.3	107
*β actin*	CACCAACTGGGACGACAT	ACAGCCTGGATAGCAACG	NM_001101.5	189

**Table 2:** Antimicrobial activity as measured by inhibition zone diameter (millimeter).

Strain	CM	CM-EXOs	CN	MCZ
*S. aureus*	11	NiL	18	-
*E. faecalis*	16	NiL	12	-
*M. luteus*	27	NiL	18	-
*E. coli*	NiL	NiL	14	-
*P. mirabilis*	NiL	NiL	15	-
*P. aeruginosa*	NiL	NiL	10	-
*C. albicans*	NiL	NiL	-	10

NiL, no antimicrobial activity recorded; CN, Gentamicin 10 mcg (standard antibiotic disc), MCZ, Miconazole 10 mcg (standard antifungal disc).

**Table 3:** CFU reduction (%) of bacterial strain cells.

Strain	CM	CM-EXOs
*S. aureus*	100.00 ± 2.28 ^a^	93.73 ± 1.74 ^b^
*E. faecalis*	100.00 ± 2.35 ^a^	91.97 ± 2.06 ^b^
*M. luteus*	100.00 ± 2.10 ^a^	90.87 ± 2.23 ^b^
*E. coli*	82.95 ± 1.64 ^b^	97.69 ± 1.49 ^a^
*P. mirabilis*	79.15 ± 1.17 ^b^	95.5 ± 2.20 ^a^
*P. aeruginosa*	75.51 ± 1.09 ^b^	90.50 ± 2.39 ^a^
*C. albicans*	83.34 ± 1.50 ^a^	68.39 ± 1.27 ^b^

Data were presented as % mean ± standard error of the mean (SEM), *n* = 9/group. Values in the same row and carrying different superscript letters (a is the highest value) are significantly different at *p* ≤ 0.05.

## Data Availability

The data presented in this study are available on request from the corresponding author.

## Supplementary Materials

The following supporting information can be downloaded at: https://www.mdpi.com/article/10.3390/vetsci10020124/s1.
