# Supplementary material for: Antibacterial, Antifungal, and Anticancer Effects of Camel Milk Exosomes: An In Vitro Study
Source: Vet Sci. 2023 Feb 6;10(2):124. doi: 10.3390/vetsci10020124 (PMC9963947; doi:10.3390/vetsci10020124)
Supplement: Supplementary file 1 [file vetsci-10-00124-s001.zip › vetsci-2016671-supplementary.pdf]

veterinary sciences

MDPI

Supplementary file shows raw data of qPCR

(1) Bax gene in HepG2 cells

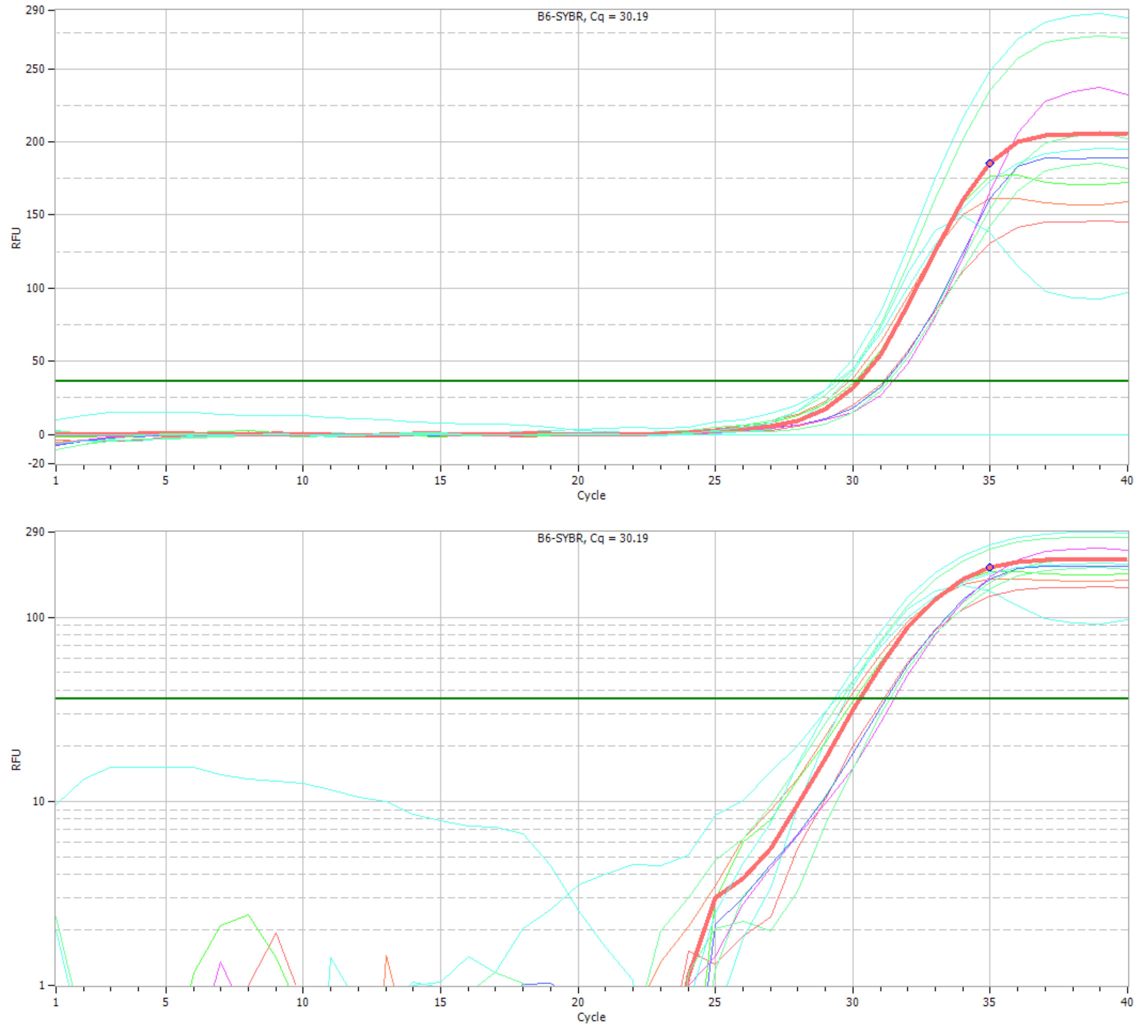

Linear (upper) and log (lower) views of the amplification curves representing the Ct values of *Bax*.

Calculation of fold change (Aver = Average of 3 replicates)

Group	*Bax* Aver CT	Delta Ct	Delta delta Ct	Fold change	SEM
Cnt	31.07	6.68	0.00	1.00	0
EXO-L	30.81	6.19	-0.49	1.40	0.07
EXO-M	30.01	5.58	-1.10	2.14	0.1
EXO-H	29.27	4.77	-1.91	3.76	0.16

(2) Bax gene in CaCo2 cells

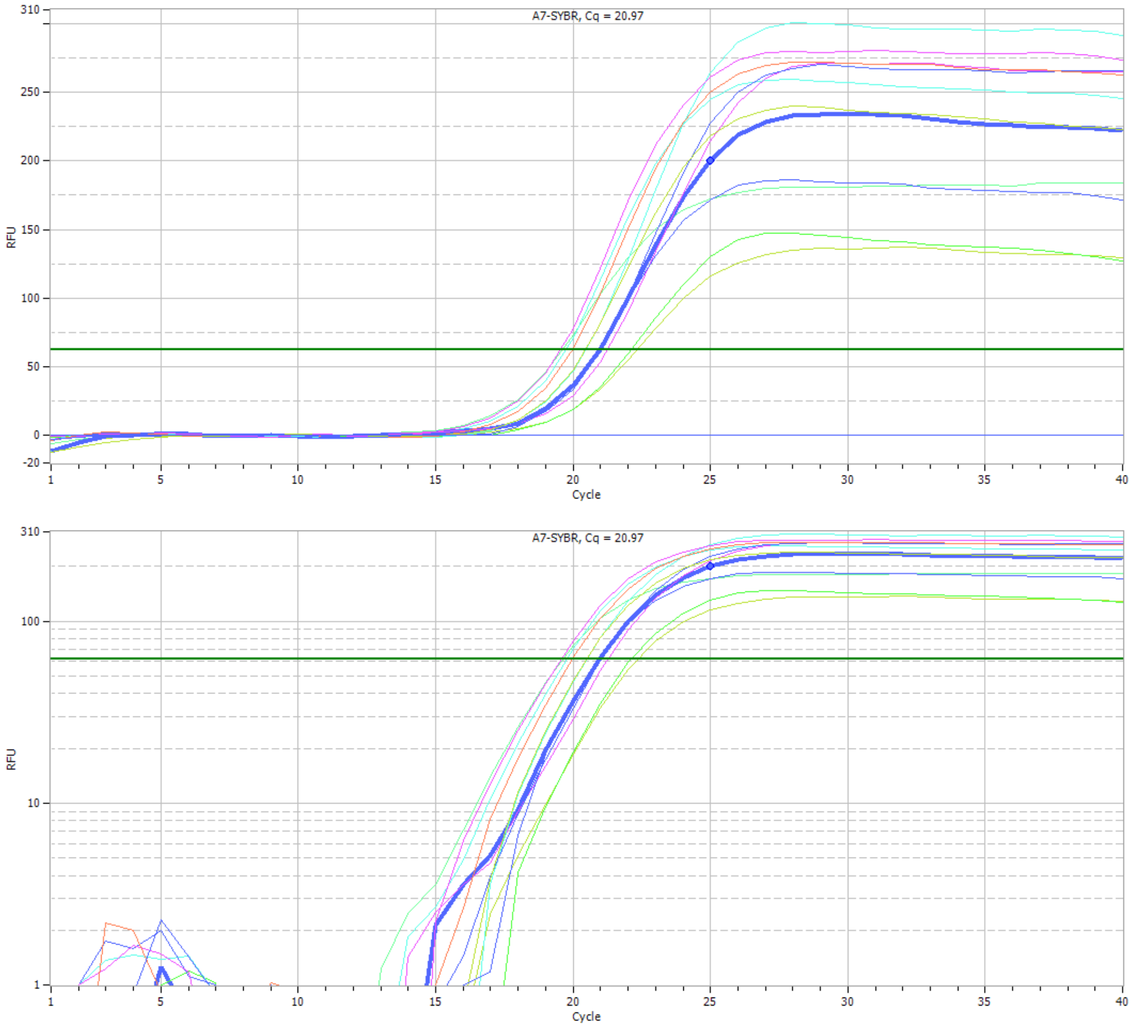

Linear (upper) and log (lower) views of the amplification curves representing the Ct values of *Bax*.

Calculation of fold change

Group	*Bax* Aver CT	Delta Ct	Delta delta Ct	Fold change	SEM
Cnt	21.84	-2.55	0.00	1.00	0
EXO-L	20.85	-3.51	-0.96	1.95	0.12
EXO-M	19.13	-5.00	-2.45	5.46	0.2
EXO-H	18.96	-5.40	-2.85	7.21	0.38

(3) Bax gene in Vero cells

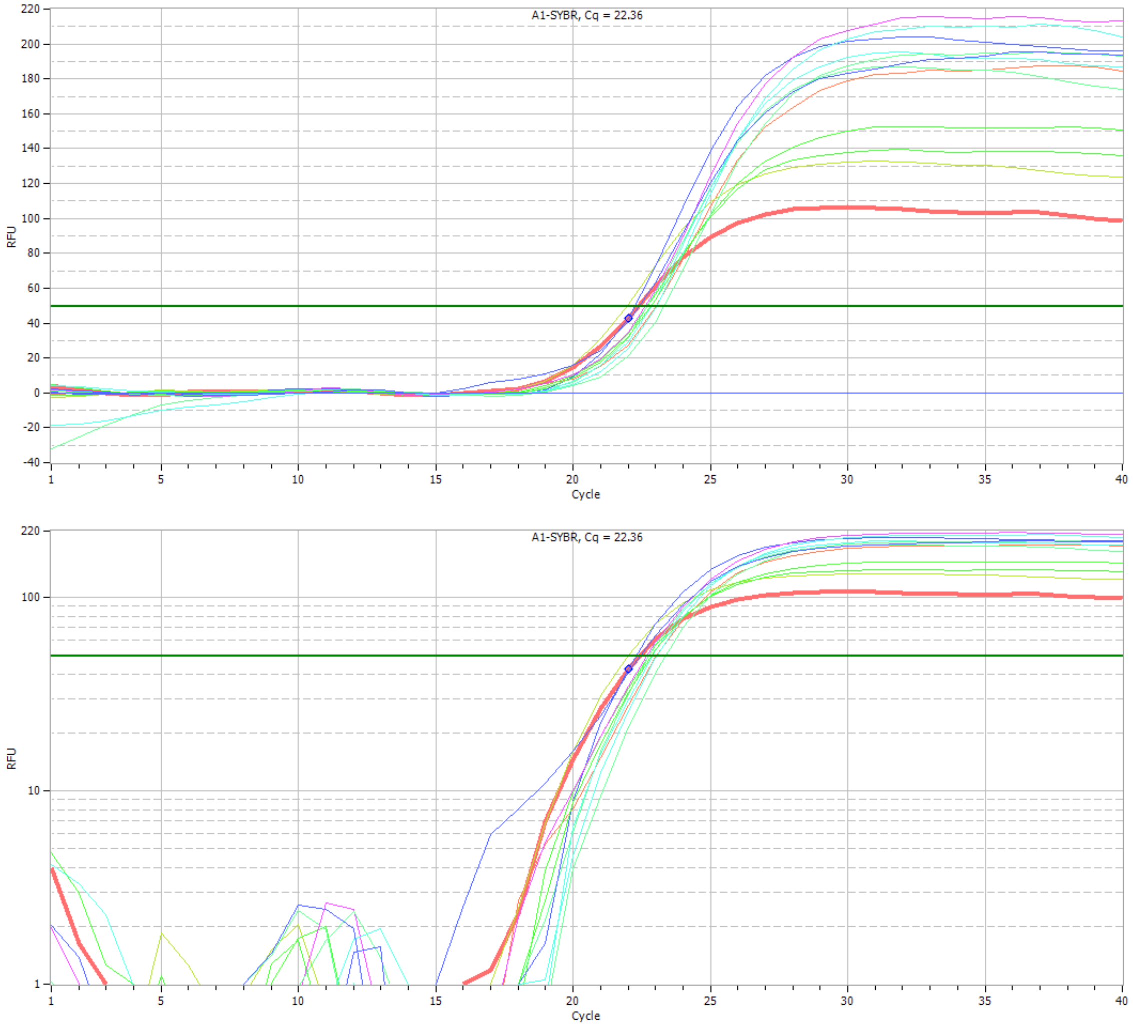

Linear (upper) and log (lower) views of the amplification curves representing the Ct values of *Bax*.

Calculation of fold change

Group	*Bax* Aver CT	Delta Ct	Delta delta Ct	Fold change	SEM
Cnt	21.05	-3.34	0.00	1.00	0
EXO-L	21.17	-3.25	0.09	0.94	0.12
EXO-M	21.8	-3.05	0.29	0.82	0.1
EXO-H	22.08	-2.80	0.54	0.69	0.19

(4) Bcl2 gene in HepG2 cells

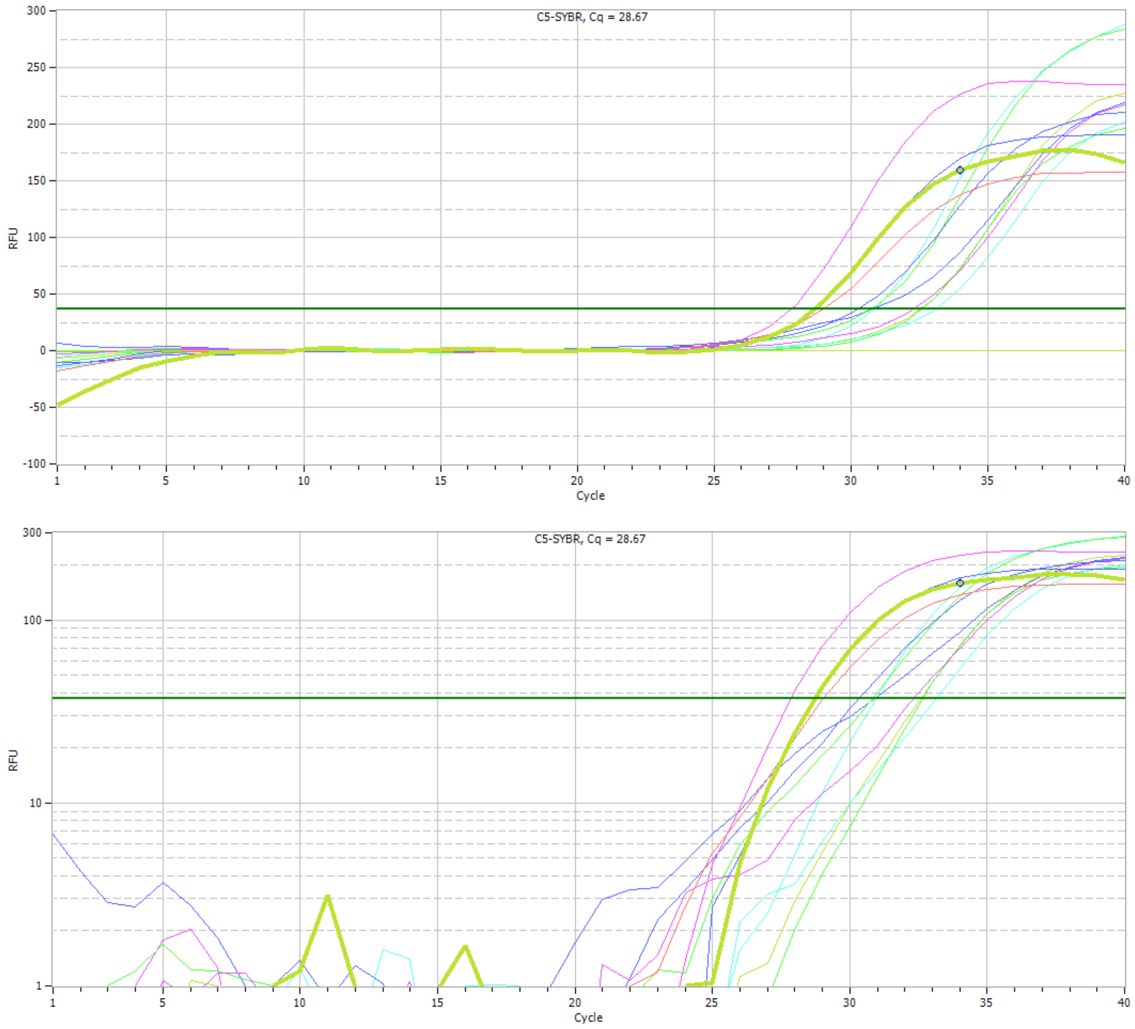

Linear (upper) and log (lower) views of the amplification curves representing the Ct values of *Bcl2*.

Calculation of fold change

Group	*Bcl2* Aver CT	Delta Ct	Delta delta Ct	Fold change	SEM
Cnt	30.66	6.27	0.00	1.00	0
EXO-L	31.56	7.24	0.97	0.51	0.04
EXO-M	31.96	7.83	1.56	0.34	0.0
EXO-H	32.59	8.34	2.07	0.24	0.01

(5) Bcl2 gene in CaCo2 cells

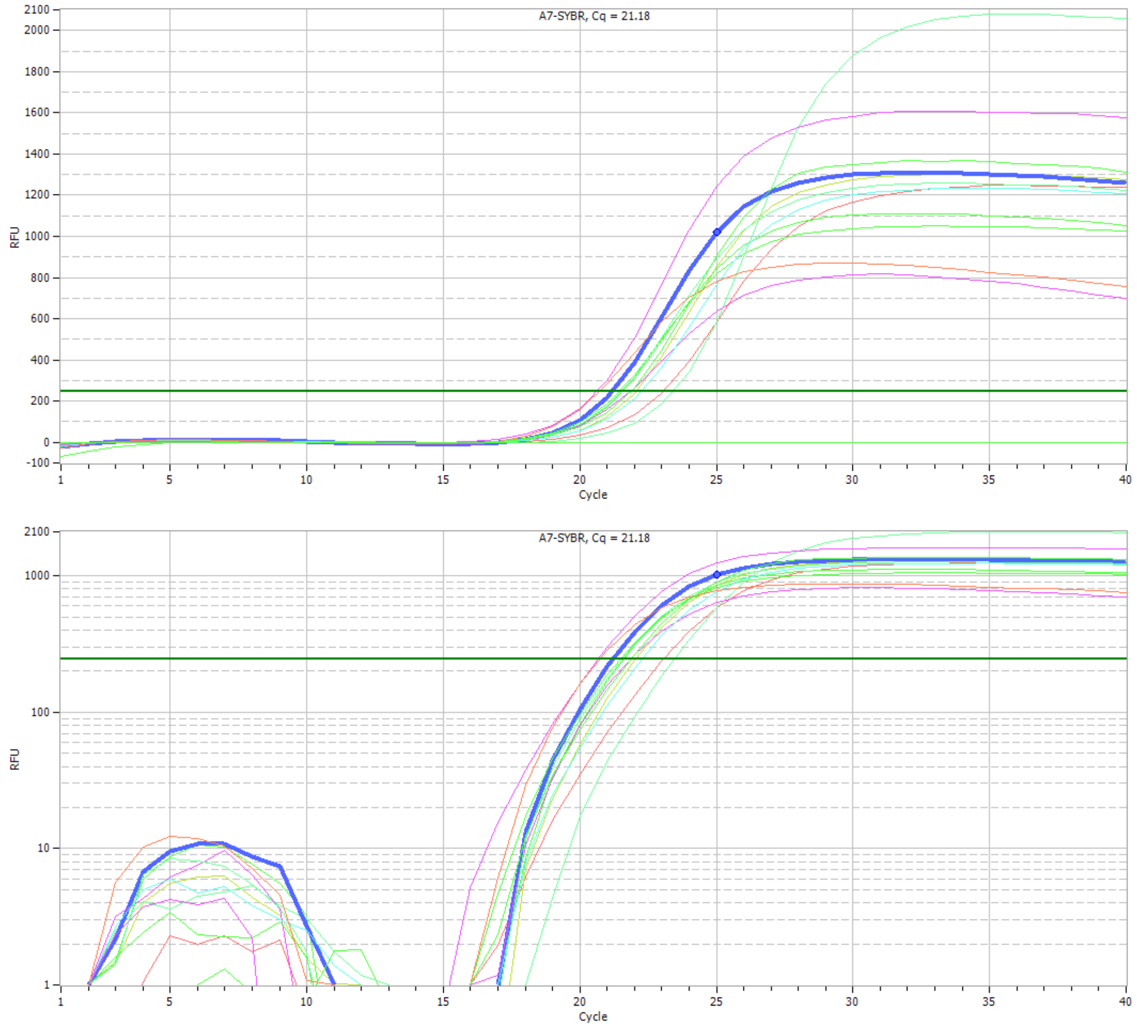

Linear (upper) and log (lower) views of the amplification curves representing the Ct values of *Bcl2*.

Calculation of fold change

Group	*Bcl2* Aver CT	Delta Ct	Delta delta Ct	Fold change	SEM
Cnt	19.03	-5.36	0.00	1.00	0
EXO-L	20.3	-4.16	1.20	0.44	0.02
EXO-M	20.52	-3.81	1.55	0.34	0.0
EXO-H	21.44	-2.92	2.44	0.18	0.01

(6) Bcl2 gene in Vero cells

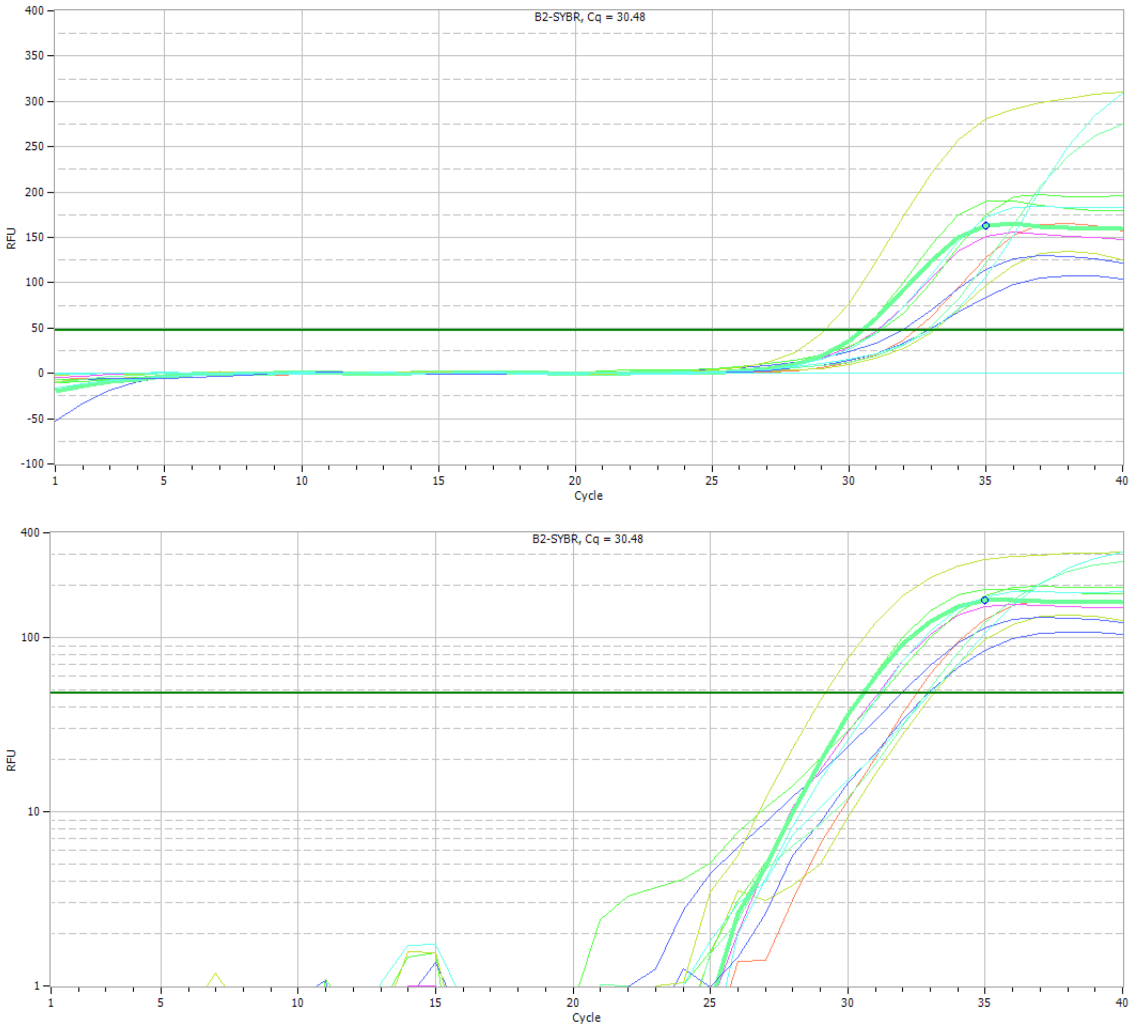

Linear (upper) and log (lower) views of the amplification curves representing the Ct values of *Bcl2*.

Calculation of fold change

Group	*Bcl2* Aver CT	Delta Ct	Delta delta Ct	Fold change	SEM
Cnt	31.2	6.81	0.00	1.00	0
EXO-L	32.08	7.22	0.41	0.75	0.15
EXO-M	31.96	7.03	0.22	0.86	0.1
EXO-H	32.38	7.42	0.61	0.66	0.22

(7) NrF2 gene in HepG2 cells

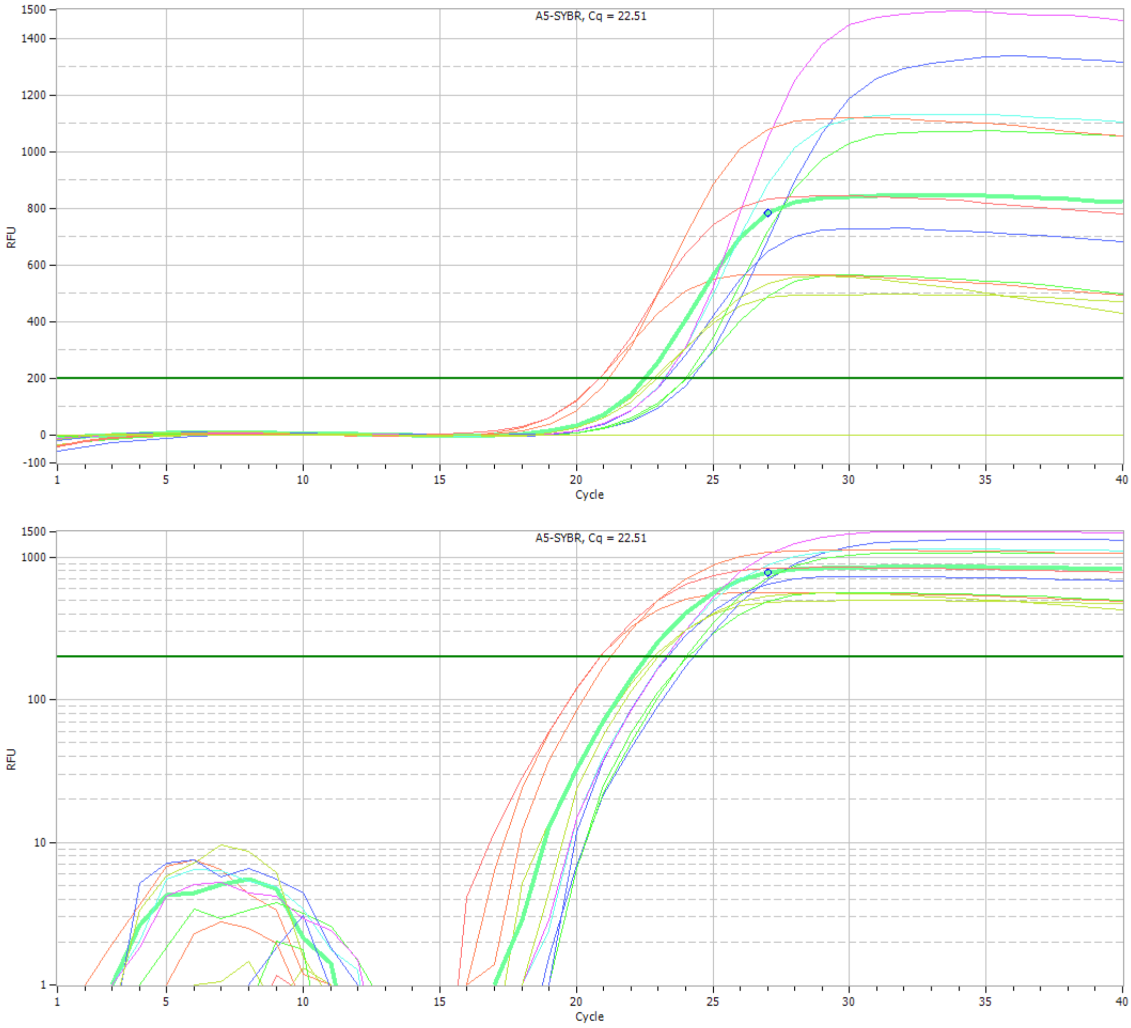

Linear (upper) and log (lower) views of the amplification curves representing the Ct values of *NrF2*.

Calculation of fold change

Group	*NrF2* Aver CT	Delta Ct	Delta delta Ct	Fold change	SEM
Cnt	20.51	-3.88	0.00	1.00	0
EXO-L	21.07	-3.77	0.11	0.93	0.06
EXO-M	21.54	-2.67	1.21	0.43	0.0
EXO-H	23.5	-1.13	2.75	0.15	0.01

(8) NrF2 gene in CaCo2 cells

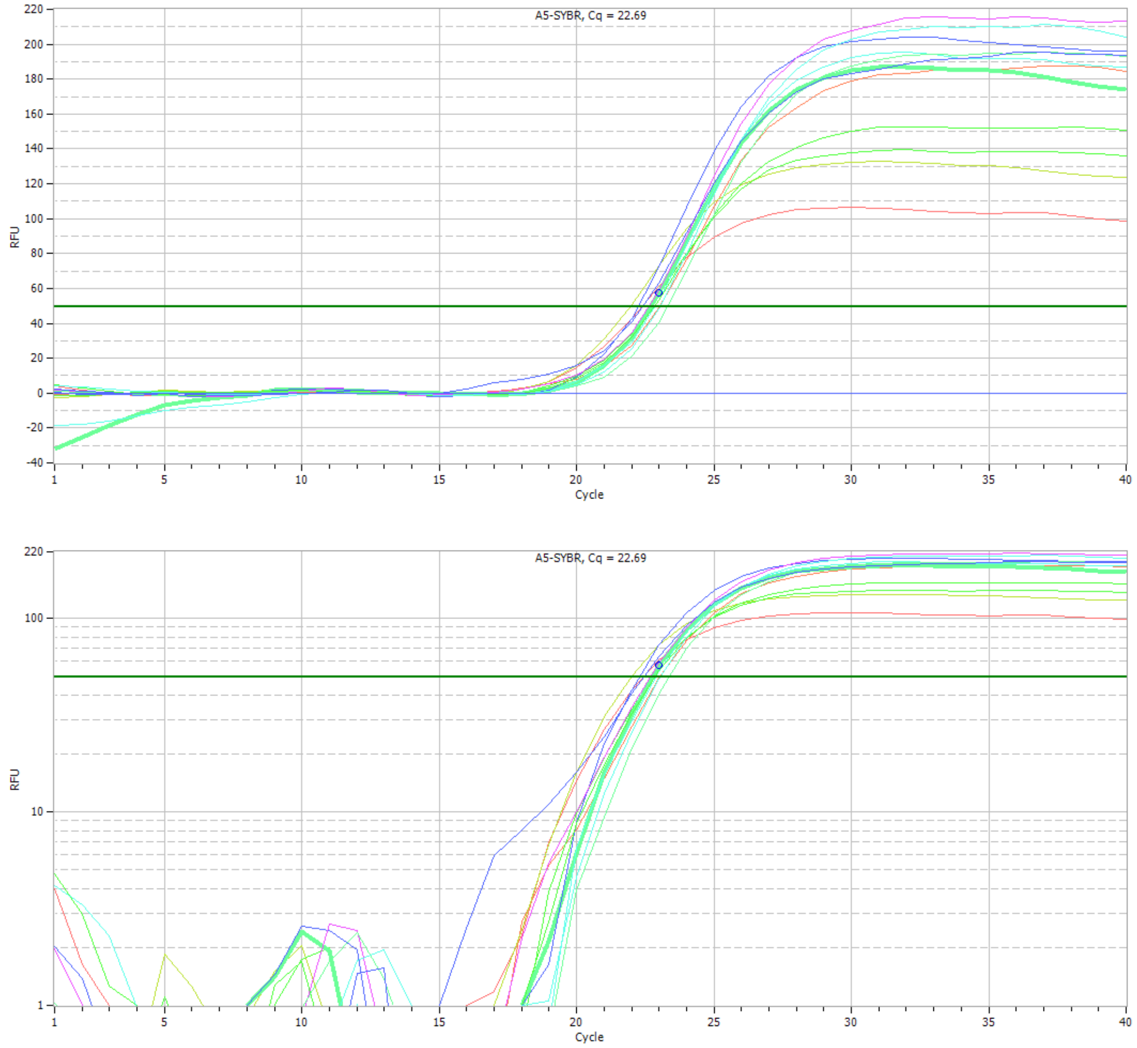

Linear (upper) and log (lower) views of the amplification curves representing the Ct values of *NrF2*.

Calculation of fold change

Group	*NrF2* Aver CT	Delta Ct	Delta delta Ct	Fold change	SEM
Cnt	20.34	-4.05	0.00	1.00	0
EXO-L	21.34	-3.50	0.55	0.68	0.03
EXO-M	22.35	-1.86	2.19	0.22	0.0
EXO-H	23.95	-0.68	3.37	0.10	0.01

(9) NrF2 gene in Vero cells

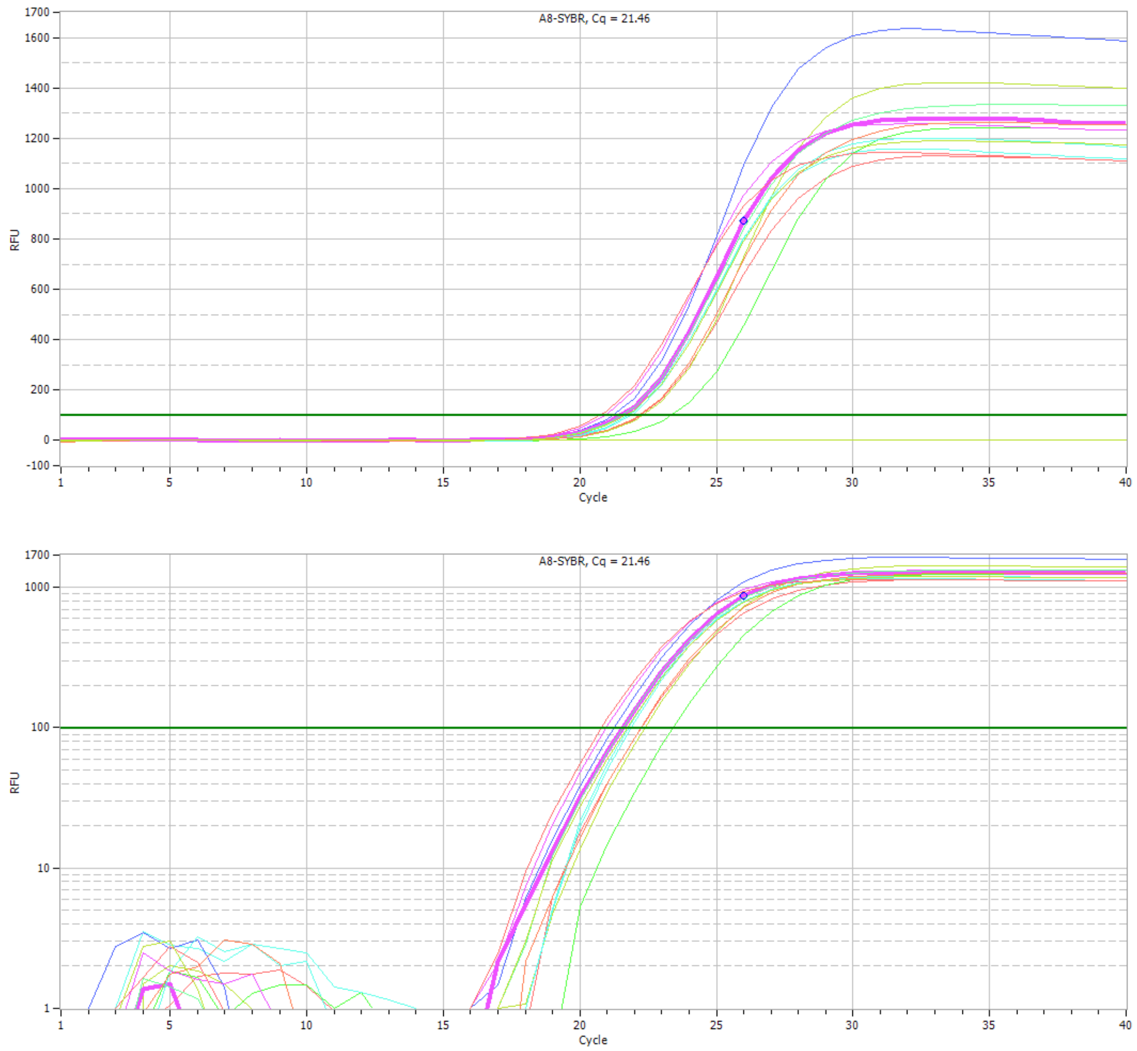

Linear (upper) and log (lower) views of the amplification curves representing the Ct values of *NrF2*.

Calculation of fold change

Group	*NrF2* Aver CT	Delta Ct	Delta delta Ct	Fold change	SEM
Cnt	20.21	-4.18	0.00	1.00	0
EXO-L	21.39	-3.45	0.73	0.60	0.21
EXO-M	21.34	-3.57	0.61	0.66	0.2
EXO-H	20.32	-4.01	0.17	0.89	0.19

(10) HO-1 gene in HepG2 cells

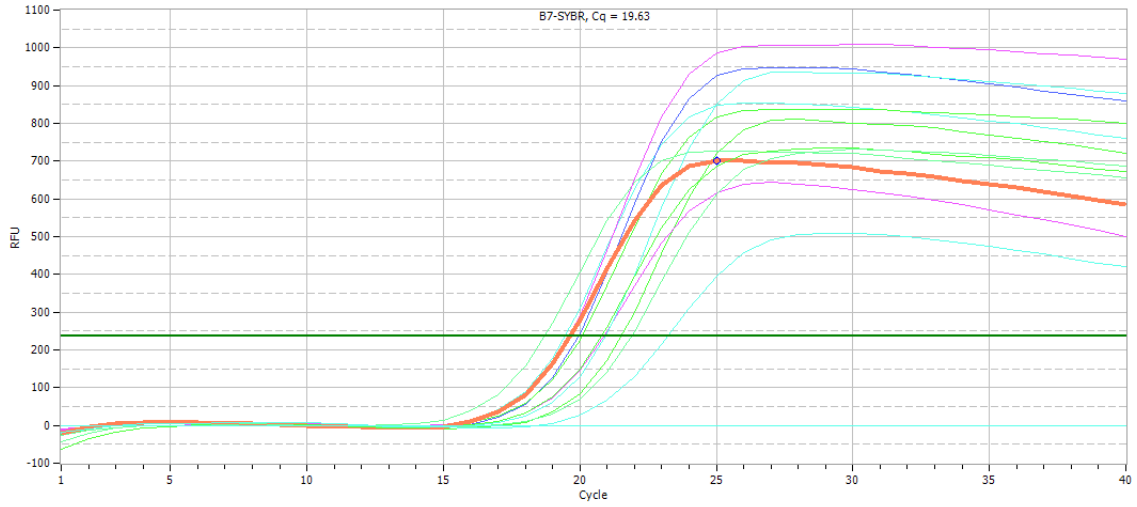

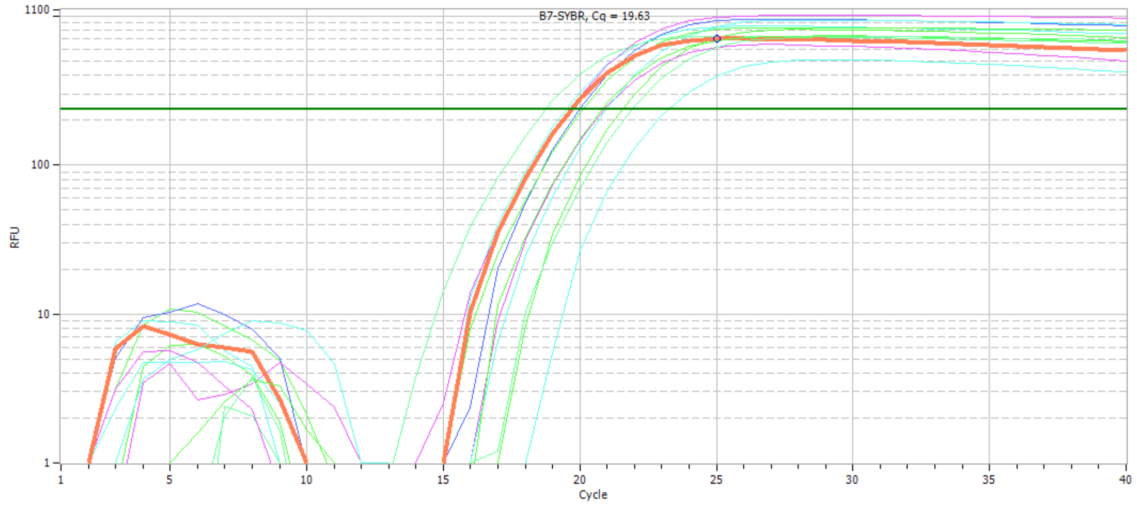

Linear (upper) and log (lower) views of the amplification curves representing the Ct values of *HO-1*.

Calculation of fold change

Group	*HO-1* Aver CT	Delta Ct	Delta delta Ct	Fold change	SEM
Cnt	18.58	-5.81	0.00	1.00	0
EXO-L	20.96	-3.70	2.11	0.23	0.01
EXO-M	21.06	-3.56	2.25	0.21	0.0
EXO-H	22.15	-2.44	3.37	0.10	0.00

(11) HO-1 gene in CaCo2 cells

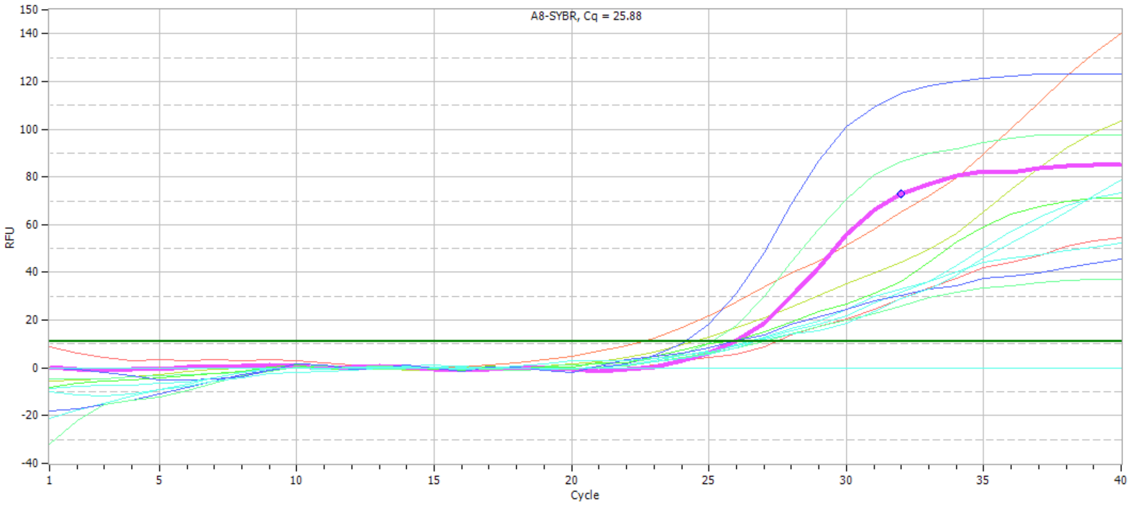

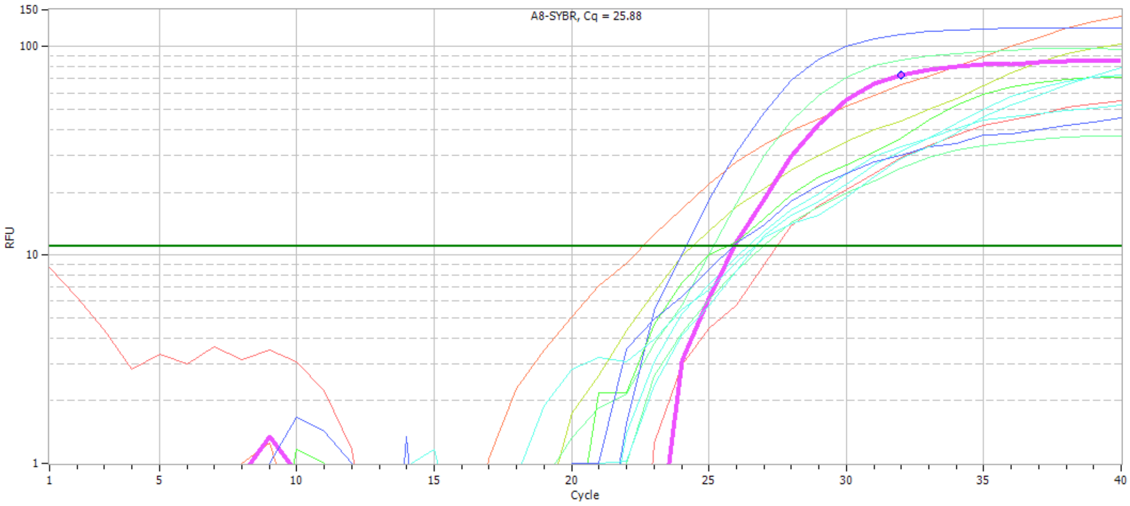

Linear (upper) and log (lower) views of the amplification curves representing the Ct values of *HO-1*.

Calculation of fold change

Group	*HO-1* Aver CT	Delta Ct	Delta delta Ct	Fold change	SEM
Cnt	25.18	0.79	0.00	1.00	0
EXO-L	26.25	1.59	0.80	0.57	0.03
EXO-M	26.5	1.88	1.09	0.47	0.0
EXO-H	27.5	2.71	1.92	0.26	0.02

(12) HO-1 gene in Vero cells

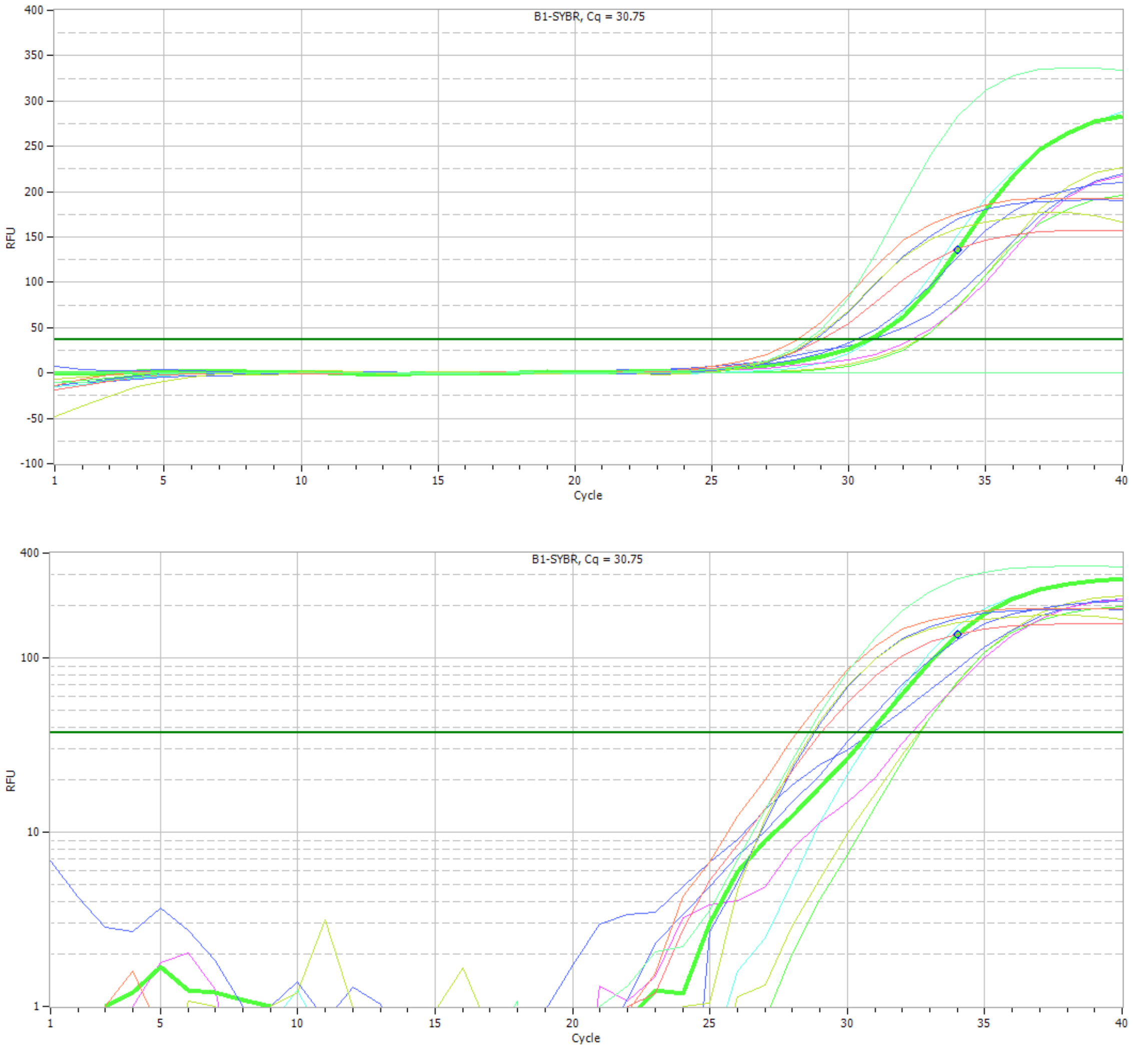

Linear (upper) and log (lower) views of the amplification curves representing the Ct values of *HO-1*.

Calculation of fold change

Group	*HO-1* Aver CT	Delta Ct	Delta delta Ct	Fold change	SEM
Cnt	31.07	6.68	0.00	1.00	0
EXO-L	30.89	6.63	-0.05	1.04	0.13
EXO-M	30.4	6.38	-0.30	1.23	0.1
EXO-H	29.95	6.25	-0.43	1.35	0.24
